# Observational and clinical evidence that plant-based nutrition reduces dietary acid load

**DOI:** 10.1017/jns.2022.93

**Published:** 2022-10-31

**Authors:** Maximilian A. Storz, Alvaro L. Ronco, Luciana Hannibal

**Affiliations:** 1 Department of Internal Medicine II, Center for Complementary Medicine, Freiburg Medical Center – Faculty of Medicine, University of Freiburg, Hugstetterstraße 55, Freiburg 79106, Germany; 2 Unit of Oncology and Radiotherapy, Pereira Rossell Women's Hospital, Bvard. Artigas 1590, Montevideo 11600, Uruguay; 3 School of Medicine, CLAEH University, Prado and Salt Lake, Maldonado 20100, Uruguay; 4 Biomedical Sciences Center, University of Montevideo, Puntas de Santiago 1604, Montevideo 11500, Uruguay; 5 Laboratory of Clinical Biochemistry and Metabolism, Department of General Pediatrics, Adolescent Medicine and Neonatology, Faculty of Medicine, Medical Center, University of Freiburg, Freiburg 79106, Germany

**Keywords:** Dietary acid load, Net endogenous acid production, Plant-based diet, Potential renal acid load, Vegan diet, Vegetarian diet

## Abstract

Contemporary diets in Western countries are largely acid-inducing and deficient in potassium alkali salts, resulting in low-grade metabolic acidosis. The chronic consumption of acidogenic diets abundant in animal-based foods (meats, dairy, cheese and eggs) poses a substantial challenge to the human body's buffering capacities and chronic retention of acid wherein the progressive loss of bicarbonate stores can cause cellular and tissue damage. An elevated dietary acid load (DAL) has been associated with systemic inflammation and other adverse metabolic conditions. In this narrative review, we examine DAL quantification methods and index observational and clinical evidence on the role of plant-based diets, chiefly vegetarian and vegan, in reducing DAL. Quantitation of protein and amino acid composition and of intake of alkalising organic potassium salts and magnesium show that plant-based diets are most effective at reducing DAL. Results from clinical studies and recommendations in the form of expert committee opinions suggest that for a number of common illnesses, wherein metabolic acidosis is a contributing factor, the regular inclusion of plant-based foods offers measurable benefits for disease prevention and management. Based on available evidence, dietary shifts toward plant-based nutrition effectively reduces dietary-induced, low-grade metabolic acidosis.

## Introduction

Contemporary diets in Western countries are largely acid-inducing and deficient in potassium alkali salts^(1,2)^. This results in a chronic condition known as low-grade metabolic acidosis, subsequent to an increased dietary acid load (DAL) that leads to small net increases in acid (H^+^) and a reduction in base (HCOO_3_^−^). While diet-induced low-grade metabolic acidosis results in only a slight decrease in blood pH, investigations that followed the initial seminal findings of Kurtz *et al.* have shown that its impact on metabolism can contribute to the worsening of a variety of disorders^(3)^. DiNicolantonio and O'Keefe have classified low-grade metabolic acidosis as a driver of chronic disease^(4)^.

In general, foods of animal origin contain precursors that increase DAL (main precursors of acid include proteins rich in sulphur-containing amino acids, lysine, and arginine), whereas the vast majority of plant-based foods are precursors of base (potassium alkali salts and magnesium). Thus, low-grade metabolic acidosis is frequently found in individuals adhering to contemporary omnivorous Western diets^(4)^. Although there are no clinically apparent or noticeable harms, the chronic retention of acid and the progressive loss of bicarbonate stores can cause cellular and tissue damage. The long-term intake of supraphysiological loads of acid in contemporary net acid-producing diets has been associated with systemic inflammation and other adverse metabolic conditions^(2,5,6)^.

The human body is naturally equipped with multiple systems to buffer and titrate acid in order to prevent the inexorable accumulation of acid^(7)^. However, the body's capacities are limited and may be insufficient under certain circumstances (e.g. in age-related decline in renal functional)^(2)^.

**Fig. 1.**
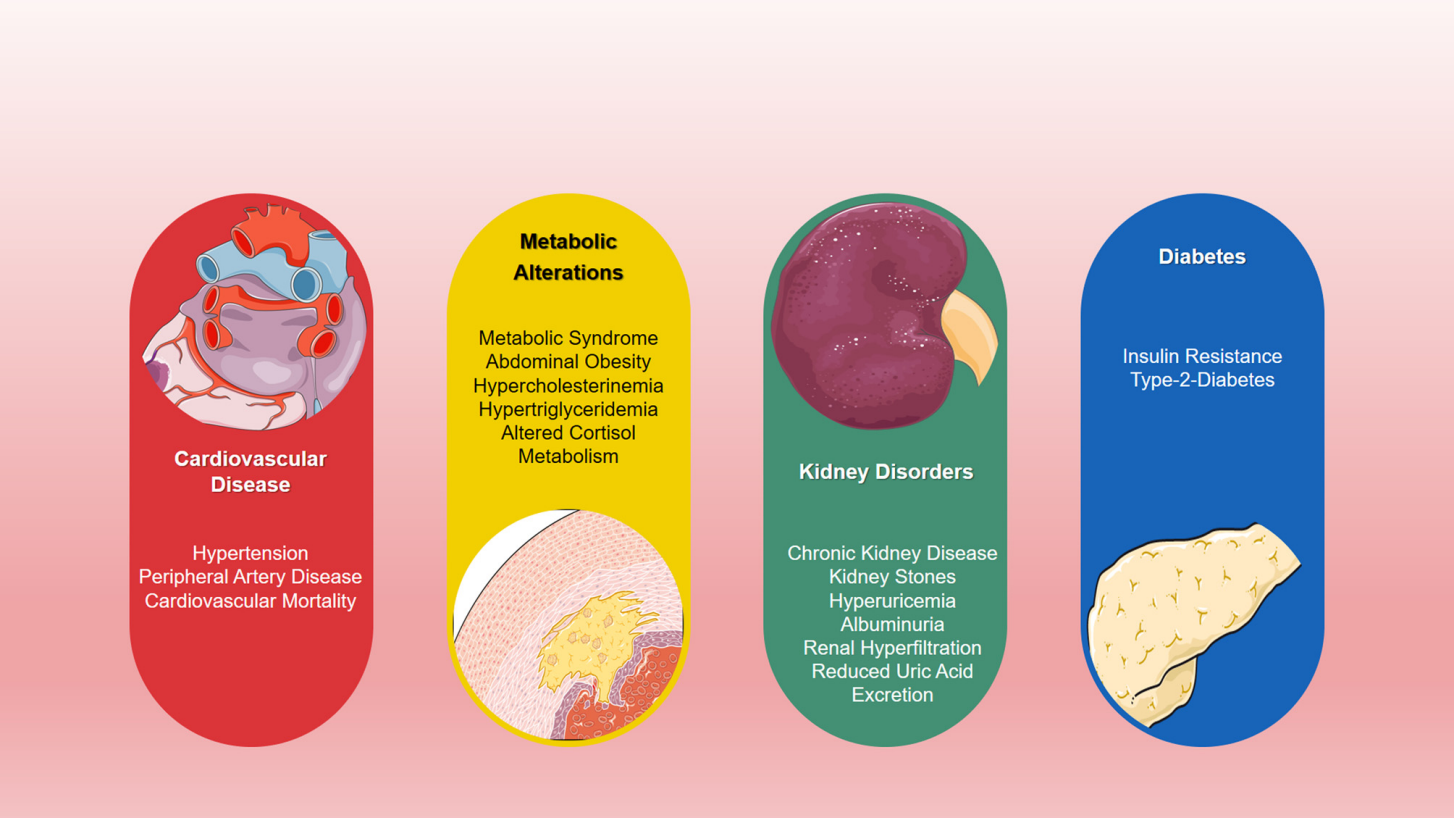
Potential adverse effects of a high DAL: an overview. Based on^(5,6,11)^. DAL, dietary acid load.

Contemporary Western diets typically produce a total acid load of about 60–100 mEq/d^(8,9)^. However, even in healthy adults, the kidneys can only excrete 40–70 mEq of acid per day before acid is retained in the body^(4)^. When acid production exceeds its excretion, compensatory mechanisms (such as muscle and connective tissue breakdown to eliminate protons along with ammonium^(4)^) are elicited to minimise systemic acidosis. This chronic acid-related stress is increasingly understood as a continuum, which has chronic metabolic acidosis at its most extreme end, and acidifying diets at its least extreme, yet also detrimental, end^(10)^. Chronic acid-stress has been associated with numerous health repercussions (Fig. 1)^(5,6,11)^.

Dietary modifications are an effective means to reduce the burden of alimentary acid load^(12)^. A frequent consumption of acid-inducing foods (processed meats, cheese and certain acidifying grains) combined with a low intake of base-inducing foods (fruits, legumes and vegetables) increases DAL^(13–15)^. Plant-based diets (PBDs) that are naturally low in (or exclude) animal products have been shown to reliably reduce DAL^(14)^. Results from clinical studies and recommendations in the form of expert committee opinions suggest that for a number of common illnesses – wherein metabolic acidosis is a contributing factor – the regular inclusion of plant-based foods offers measurable benefits for disease prevention and management^(16)^.

This review examines the contribution of plant-based dietary patterns, chiefly vegetarian and vegan diets, which drastically reduce or exclude animal products, to DAL and summarises growing evidence that dietary shifts toward plant-based nutrition are effective at diminishing dietary-induced low-grade metabolic acidosis.

### DAL assessment and quantification

Epidemiological studies and clinical trials regularly rely on estimates of DAL to investigate potential relationships to human health and disease^(17)^. The majority of studies on DAL used at least two common formulas to estimate acid load from diet: the potential renal acid load (PRAL) score by Remer and Manz^(18)^ and the net endogenous acid production (NEAP) score by Frassetto *et al.*^(19)^.

The PRAL score may be calculated as follows^(18)^:
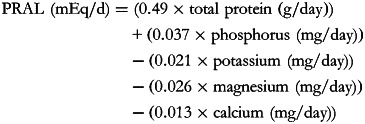


The PRAL score (hereafter called PRAL_R_) includes intestinal absorption rates for the following micronutrients: potassium, phosphate, magnesium, calcium and also considers protein intake. Previous studies in healthy individuals validated a strong correlation between the PRAL score and urinary pH^(18)^.

NEAP (hereafter called NEAP_F_) may be estimated based on the formula by Frasetto *et al.*^(19)^, which considers daily total protein intake and potassium intake.
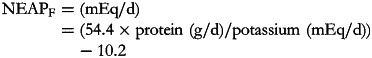


Alternatively, there is an additional formula to estimate NEAP proposed by Remer *et al.* (hereafter termed NEAP_R_)^(20)^.
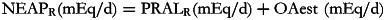


Remer *et al.* estimated NEAP from average intestinal absorption rates of ingested protein and additional minerals (PRAL_R_ score) as well as anthropometry-based estimates for organic acid excretion (OAest). Hereby, OAest (mEq/d) was calculated as follows:
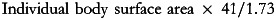


The individual body surface area may be calculated with the formula of Du Bois and Du Bois:
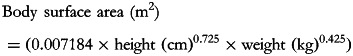


NEAP is the net combination of non-carbonic (i.e. fixed) acids from acids ingested in the diet and produced from endogenous metabolic processes, minus the acids that are neutralised or buffered by non-carbonic dietary and endogenously generated base supplies^(21,22)^. Thus, NEAP considers PRAL (e.g. the sum of bases and acids released from diet-derived compounds of cations and anions) *in addition* to the total non-carbonic organic acids. The aforementioned NEAP scores (NEAP_F_, NEAP_R_) have both been validated against net acid excretion (NAE) with satisfying results, reliably estimating NAE).

The three aforementioned scores (NEAP_F_, NEAP_R_ and PRAL_R_) are the most commonly used scores in the majority of studies. Notably, each score has its own advantages and drawbacks^(23)^. In clinical practice, all scores performed differently^(21)^. Calculation of all three scores is thus recommended, preferably supplemented with estimations of urinary DAL indices (PRAL and NEAP) values, as recently summarised by Parmenter *et al.*^(22)^.

Western diets typically produce a total DAL ranging from approximately 50 to 75 mEq/d^(8,9)^. In other parts of the world, DAL is substantially lower. One is rural Ghana, where Goldberg *et al.* reported NAE values of 29⋅2 ± 12⋅2 mEq/d^(24)^. When glancing solely at PRAL values, there are notable differences between common dietary patterns^(10)^. Wesson reported calculated PRAL sums of selected diets and demonstrated that the average dietary intake in the United States results in PRAL sums of approximately 27 mEq/d. Other diets, such as the DASH diet (Dietary Approaches to Stop Hypertension) resulted in substantially lower PRAL sums (about 11 mEq/d). PBDs are characterised by even lower PRAL sums^(12)^. The potential DAL-lowering mechanism of PBDs is discussed in detail hereafter.

### Dietary components affecting DAL

The ratio of plant *v*. animal-based food intake determines DAL^(25)^. When protein containing foods are metabolised, most release acid in the form of hydrogen ions. In contrast, potassium-rich plant foods (mainly fruits and vegetables) produce alkali^(25,26)^.

### Protein content and amino acid composition of diet

Unlike carbohydrates or lipids, which do not generate unmetabolisable acidity during their complete oxidation, proteins contain various amino acids whose catabolism is liable to affect the acid–base equilibrium^(1)^. When protein containing foods are metabolised, most release acid in the form of protons^(25)^. The amount, however, depends on the amino acid composition. Some amino acids are neutral, some are acidic and some are alkaline^(25,27)^.

Lysine, arginine and histidine are acidifying because their metabolisation in the liver generates hydrochloric acid (plus glucose and urea)^(1,25)^:
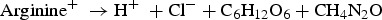


Lysine and arginine intake are substantially higher on a meat-based diet as compared to plant-based (vegan) diet^(28,29)^. While this might be beneficial with regard to DAL, it has also been argued that an insufficient lysine intake could also have adverse effects on human health. Yet, if a diet has at least a modest amount of variability (which is usually the case in economically developed countries), there are no issues regarding sufficient intakes of lysine^(30)^.

Another group that belongs to the acidifying amino acids is sulphur-containing amino acids (methionine, homocysteine and cysteine)^(31)^. Catabolism of these amino acids leads to sulphuric acid generation – a non-metabolizable anion which is a major constituent of DAL^(25,27)^.

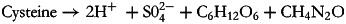


The obtained sulphate anions constitute unmetabolizable acidity^(1)^ and are a major contributor to DAL^(32)^. Plant-based proteins tend to be much lower in methionine than animal proteins^(33,34)^. As summarised by McCarty, the methionine fraction in representative plant proteins ranges from 0⋅85 % in lentils to 2⋅26 % in brown rice, whereas that of animal proteins falls into a much higher range (from approximately 2⋅35 to 3⋅11 %)^(33)^. Eggs are often high in methionine^(35)^, whereas the fraction of methionine in legume protein and nut protein is especially low^(36)^. Table 1 summarises the methionine content of selected common foods per kcal (based on^(33)^). For additional information on amino acid composition in selected foods across foods groups, we refer the interested reader to the work of Gardner *et al.*^(37)^.

**Table 1.** Content of the amino acid methionine in commonly consumed foods of plant and animal origin

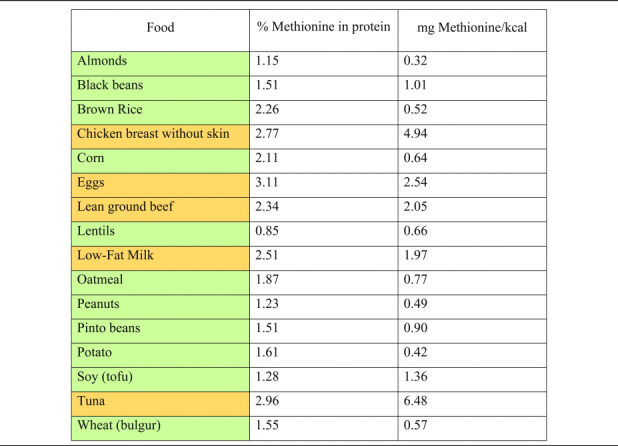

Orange colouring: animal-based foods, green colouring: plant-based foods.

Source data adapted from^(33)^.

In this context, mentioning of the amino acid glycine is also warranted. Glycine can act as a functional Methionine antagonist^(33)^, since it can fulfil the role of a methyl group acceptor in a biochemical reaction catalysed by glycine *n*-methlytransferase – a key enzyme in methyl group metabolism^(38)^. Plant proteins are higher in glycine than most animal proteins^(39)^ and it is not surprising that vegans had the highest plasma concentration of this amino acid in the Epic Oxford cohort study^(28)^.

#### Glutamate and glutamine content of diet

Glutamine (C_5_H_10_N_2_O_3_) and glutamate (C_5_H_9_NO_4_) are important for the neutralisation of acid via α-Ketoglutaric acid (C_5_H_6_O_5_). Glutamate is a non-essential neutralising anionic amino acid whose metabolism consumes hydrogen ions to become neutral^(25,27,40)^:
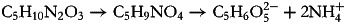

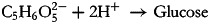


Diet is the major source of glutamine and glutamate^(41)^, and unprocessed plant proteins are usually richer than animal proteins in glutamate^(27)^. In cross-sectional studies, meat eaters thus had a lower glutamine intake than vegetarians and vegans^(28)^. Another prominent example with comparable findings is the INTERMAP study, demonstrating that individuals on a high plant protein/low animal protein diet consumed greater amounts of glutamic acid as compared with their high animal protein/low plant protein counterparts^(42)^. A reservation must be made that this section refers to unprocessed plant foods and not to processed vegan foods enriched with artificial flavours containing monosodium glutamate.

#### Phosphorus content of diet

Phosphorus and preservative phosphates (phosphoric acid, polyphosphates, etc.) are other important contributors to DAL^(14)^. Phosphate salts are frequently added to bacon, sausages and other processed meats for their antibacterial properties and to condition the colour and flavour of products^(43,44)^. In addition to that, phosphate additives are frequently found in cheese manufacture and milk products^(45,46)^.

Notably, their acidity does not depend on the phosphate anion itself^(25)^. Instead, it depends on the cation to which the phosphate anion is attached and the pH of the food. Phosphoric acid (H_3_PO_4_), commonly found in many sodas and cola drinks, is acidic as H^+^ is released upon metabolisation^(25)^.
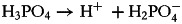


Moreover, some of the widely used preservative phosphates and additives are acidic and some are alkaline^(25)^. A frequently encountered acidic phosphate-based additive is calcium pyrophosphate (CaH_2_P_2_O_7_)^(25)^, which is frequently found in quick breads and sweet bakery products^(47)^. Trisodium phosphate (Na_3_PO_4_), on the other hand, is alkaline and consumes 2 H+ ions upon metabolisation.

The extra burden from phosphorus coming from processed products alone might reach up to 737 mg/d^(48)^. Glancing at the PRAL_R_ formula shows that phosphorus has the highest weighting factor of all micronutrients (0⋅037)^(18)^. An extra intake of 250 mg of phosphorus per day will increase PRAL by more than 9 mEq/d.

It is important to understand the extra ‘DAL burden’ subsequent to a high phosphorus intake. Milk and dairy products account for more than 24 % of phosphorus intake in human diets^(49)^, and phosphorus intake might increase substantially when other foods abundant in phosphate (e.g. soft drinks and canned fish) are consumed^(50–52)^. Table 2 shows the phosphorus content of selected foods^(53)^. In this context, a reservation must be made, that the intestinal absorption of phosphorus from additives used in food manufacturing is substantially higher compared with phosphorus derived from unprocessed animal-based foods. Relativisation is thus necessary when evaluating different phosphorus sources.

**Table 2.** Phosphorus and protein content of commonly consumed foods of plant and animal origin

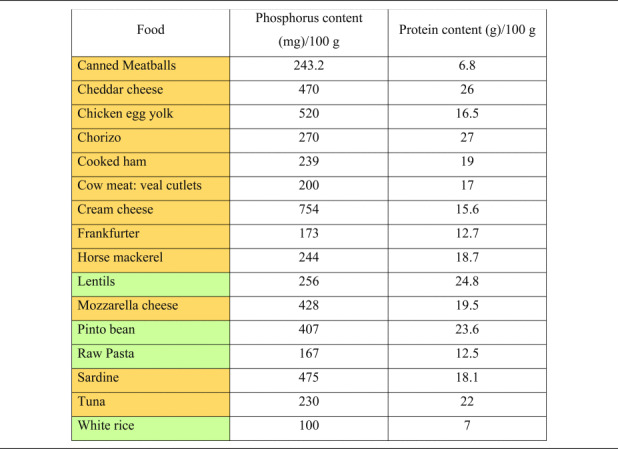

Phosphorous and protein content are expressed per 100 g of uncooked food, as typically provided in nutritional content labelling.

Orange colouring: animal-based foods, green colouring: plant-based foods.

Source data adapted from^(53)^.

Plant foods (vegetables, legumes and seeds), on the other hand, contain phosphorus in the form of phytate, which has a significantly lower bioavailability and neglectable acidising effects^(14,54,55)^. Instead, most plant-based foods have alkalising effects due to their high availability of potassium salts of organic anions^(27)^.

#### Potassium/organic anion content of diet

As a general rule, almost all fruits and vegetables display negative PRAL values, and the amount of potassium present in those foods reflects their alkalising ability^(25,56,57)^. Organic anions may be considered as virtual precursors of KHCO_3_ and can be metabolised to bicarbonate^(1,58)^.

Prominent examples include citric acid, malate and potassium citrate (C_6_H_5_K_3_O_7_)^(25)^. Organic salts such as potassium citrate contain base ions but no hydrogen ions. They are thus capable of binding hydrogen ions during their metabolism to carbon dioxide and water.
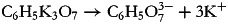

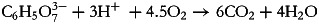


The consumption of hydrogen ions upon metabolisation has alkalising effects^(11,27)^. Except for ripened and processed grains, most plant foods contain substantial quantities of organic anions, whereas they are scarce in animal-based foods^(1)^. Daily food supply of organic anions strongly depends on dietary patterns and ranges from 1 g/d (in low plant consumers) to 3–4 g/d in a diversified omnivorous diet. Vegetarians and vegan usually consume more than 5 g/d of organic anions^(1)^. Potassium content of selected foods is presented in Table 3, based on current data from the dietary guidelines for Americans and the US Department of Agriculture^(59,60)^.

**Table 3.** Potassium and magnesium content of selected foods per standard portion

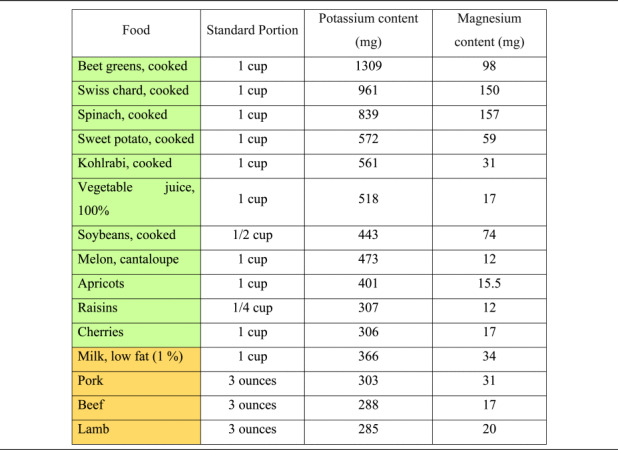

Orange colouring: animal-based foods, green colouring: plant-based foods.

Source data adapted from^(59,60)^.

Another important source of organic anions is their production in the colon, mainly short-chain fatty acids (SCFA, including butyrate, acetate and propionate)^(1)^. SCFA are the end-products of microbial fermentation in the distal part of the digestive tract, using specific substrates such as fibre and carbohydrates. SCFA production is closely dependent on nutritional factors and faecal levels of those metabolites correlate positively with the consumption of vegetables, fruits and legumes^(61)^. Significant increases in SCFA production have been observed when omnivores consume a diet rich in fruits and vegetables^(62)^, and it is now widely accepted that a plant-based vegan diet may increase SCFA production by modulation of the gut microbiota^(63,64)^.

#### Magnesium content of diet

Magnesium is a key micronutrient in the PRAL-formula by Remer and Manz, with a relatively high weighting factor of 0⋅026^(18)^. PBDs are much more abundant in magnesium than omnivorous diets^(65,66)^, and thus have a higher PRAL-lowering capacity. A Danish study revealed that vegan men consume – on average – more than 230 mg of magnesium more than the general population^(67)^, potentially translating into a PRAL-lowering capacity of more than 8 mEq/d. Magnesium content of selected foods is presented in Table 3, based on current data from the US Department of Agriculture^(59)^.

### Contribution of increased DAL to chronic illnesses

A number of studies have identified elevated DAL as a factor contributing to various chronic illnesses, such as type 2 diabetes, hyperlipidaemia, cardiometabolic disorders, renal disease, cancer and even pathologies where the metabolic component is less clear, such as mental and musculoskeletal disorders. The underlying pathomechanisms are complex and the subject of ongoing research.

Central to most chronic lifestyle-related diseases, a high DAL enhances cortisol production^(68)^, which, in turn, may promote insulin resistance^(69)^. Apart from increasing glucocorticoid secretion, a high DAL also reduces catabolic degradation of potentially bioactive glucocorticoids^(68)^. Both mechanisms ensure a steady glucocorticoid supply, which is apparently necessary to increase catabolism of skeletal muscle protein (ensuring an augmented renal glutamine supply) and the subsequent increase in renal ammoniagenesis as discussed below.

Latent metabolic acidosis subsequent to an acidifying diet may also stimulate ammoniagenesis, which allows for a simultaneous elimination of hydrogen ions and anions^(27)^. Ammoniagenesis, however, comes at its price, and has been associated with renal tubulointerstitial injury and subsequent impaired kidney function^(58,70)^. Additional adverse mechanisms include decreased uric acid excretion (potentially resulting in hyperuricaemia)^(71,72)^, increased renal excretion of calcium and magnesium^(73)^, higher insulin-like growth factor (IGF) levels^(74)^ and decreased circulating adiponectin levels through acidosis-induced inhibition of adiponectin gene transcription in adipocytes^(74,75)^.

We summarise main findings on the contribution of DAL to these diverse groups of disorders with the fundamental understanding that the causes and mechanisms of such complex illnesses are of multifactorial nature, implying that it is very likely that more than one of the aforementioned mechanisms is involved.

#### Type 2 diabetes

A high DAL has been associated with insulin resistance and an increased risk for type 2 diabetes (T2DM) in various large epidemiological cohort studies, including the Teheran Lipid and Glucose Study^(76)^, the Nurses’ Health Study I and II and the Health Professionals’ Follow-up Study^(77)^. Such associations have been found in both children/adolescents^(78)^ and adults^(79)^. A high DAL is not only associated with higher fasting blood glucose levels^(80)^ but also with impaired insulin sensitivity^(14,81)^. Notably, a high DAL may also adversely affect other clinical outcomes in individuals with T2DM. One example is a 2020 study, that demonstrated associations between higher DAL scores and impaired sleep quality and mental health disorders in said individuals^(82)^.

On the other hand, a more alkaline diet has been shown to exert protective effects^(13)^. The particular mechanisms underlying the association between metabolic acidosis and insulin resistance are yet to be elucidated. Apart from DAL-induced increased hepatic gluconeogenesis and disrupted binding of insulin to the insulin receptor, inhibition of insulin signalling pathways may play a crucial role^(13)^. These factors may play an important role when glancing at other adverse clinical outcomes related to a high DAL, including hyperlipidaemia and the increased risk for cardiometabolic disorders.

#### Hyperlipidaemia and cardiometabolic disorders

In 2008, Murakami *et al.* reported the findings of a Japanese cross-sectional study comprising 1136 female Japanese students aged 18–22 years^(83)^. The authors reported positive associations of a high DAL with higher systolic and diastolic blood pressure as well as with total and LDL-cholesterol. Associations with hypertriglyceridaemia have been reported in a cross-sectional study including 357 Iranian elderly men^(84)^. Increasing cortisol production caused by mild metabolic acidosis could be the underlying mechanism^(85)^, but additional research is warranted in this poorly understood field^(83)^. Other research suggesting potential associations between a high DAL and obesity^(86–88)^, and hypertension^(89,90)^ – where elevated cortisol levels also play an important role – support this hypothesis. Notably, a high DAL may not only increase the risk for cardiovascular disease^(91–93)^ but may also affect other organs, such as the liver (in the form of non-alcoholic fatty liver disease^(94,95)^) and the kidneys.

#### Renal disorders

Numerous clinical and epidemiological studies associated elevated DAL scores with incident chronic kidney disease^(96,97)^ and end-stage renal failure risk^(58)^. A high DAL may contribute to a faster decline in glomerular filtration rate (GFR)^(98,99)^, whereas dietary alkali treatment of metabolic disease in chronic kidney disease preserves GFR and reduce kidney angiotensin-II-activity^(100)^. Renal hyperfiltration subsequent to a high DAL^(101)^ plays a crucial role in the pathogenesis of glomerular disorders and its attenuation is considered a novel therapeutic target in diabetes and obesity-induced kidney disorders^(102)^. This again demonstrates that the effects of a high DAL are not confined to a single organ but may involve the body as a whole.

Studies on the contribution of DAL to kidney disease have gained recognition. Fruit and vegetable treatment of chronic kidney disease-related metabolic acidosis is as effective as oral NaHCO_3_ when it comes to GFR preservation but reduce cardiovascular risk better than sodium bicarbonate alone^(103,104)^. A committee of experts representing the workgroup of the Kidney Disease Outcomes Quality Initiative (KDOQI) from the National Kidney Foundation, USA, has recently published recommendations for the dietary management of DAL. These are as follows:

‘Statements on Acid Load: Dietary Management of Net Acid Production (NEAP)

In adults with CKD 1–4, we suggest reducing net acid production (NEAP) through increased dietary intake of fruits and vegetables (2C) in order to reduce the rate of decline of residual kidney function.’^(105)^

#### Musculoskeletal health and body composition

An elevated acid-load burden from dietary intakes has been associated with poor musculoskeletal health^(106,107)^ and impaired bone health^(108)^. Data from a Japanese study also suggest associations of increased DAL with frailty (particularly weakness and slowness) in older women^(109)^. Faure *et al.* reported an inverse association between PRAL and the percentage of total lean body mass among senior women in a Swiss-based population, suggesting potentially beneficial effects of a more alkaline diet in said women^(110)^. Their cross-sectional study essentially confirmed the findings by Welch *et al.*, who reported a positive association of a more alkaline PRAL with fat-free mass (%) among women between 18 and 79 years, independent of physical activity and smoking^(111)^. Notably, much additional research is warranted in this field as a recent study associated higher acid diet measures with higher muscle strength – contrary to the common acid hypothesis^(112)^.

#### Mental health

With regard to mental health, positive associations were found for depression and anxiety^(113–115)^ as well as with emotional problems and hyperactivity in children^(116)^. Systemic inflammation subsequent to a high DAL could play an important aetiological role here, yet the reservation must be made that the involved pathological mechanisms are subject to a controversial debate.

#### Cancer

Elevated DAL scores have been linked to low-grade inflammation (as indicated by elevated lipid accumulation product levels)^(84)^. It is now widely accepted that low-grade metabolic acidosis may induce peroxidation of biological structures^(1)^. An altered acid–base equilibrium may also modulate molecular activity including adrenal glucocorticoid, IGF-1 and adipocyte cytokine signalling, which contribute to dysregulated cellular metabolism and may play a role in cancer development^(74)^.

DAL-induced low-grade mild metabolic acidosis promote tissue damage and inflammation^(11,13,117,118)^, which may initiate genomic instability on normal cells through the activation of cytokines, which may stimulate tumour invasion and metastases^(119,120)^. Positive associations between a high DAL and various cancers have been reported, including breast cancer^(121,122)^, prostate cancer^(123)^, lung cancer^(124)^, colorectal cancer^(125)^, pancreatic cancer^(126)^, gastric cancer^(127)^, oesophageal cancer^(128)^ as well as head and neck cancers^(129)^. Two meta-analyses confirmed these associations: Keramati *et al.* and Bahrami *et al.* independently found higher odds for cancer in individuals with elevated DAL scores^(130,131)^.

### PBDs to reduce DAL

Dietary components affecting acid load have been discussed in detail in the previous section. PBDs, including vegetarian and vegan diets, are abundant in potassium salts of organic anions^(1,27)^, while they are at the same time low in phosphorus and preservative phosphates^(14,132)^. Although diversified PBDs contain sufficient amounts of protein, their overall content is usually lower than in omnivorous diets^(30)^. In addition to that, their content of sulphur-containing amino acids is also substantially lower as compared with meat-based diets^(33,34).^

The combination of these factors qualifies plant-based nutrition as an ideal tool to reduce DAL^(132)^. This section summarises supporting evidence for this glancing at both observational (Table 4) and clinical intervention studies (Table 5).

**Table 4.** Observational studies investigating DAL scores in plant-based cohorts

Study (year)	Location	Participants	Results	Comments
Deriemaeker *et al.*^(133)^	Belgium	*n* 60 participants, thereof *n* 30 vegetarians and *n* 30 non-vegetarians matched for age, sex and BMI	PRAL_R_: −10⋅9 ± 19⋅7 in vegetarians and 13⋅8 ± 17⋅1 in non-vegetarians NEAP_R_: 31⋅4 ± 21⋅4 in vegetarians and 56⋅4 ± 21⋅2 in non-vegetarians	NEAP_F_ was not determined as part of the study
Ströhle *et al.*^(23)^	Germany	*n* 154 participants, thereof *n* 56 moderate vegans and *n* 98 strict vegans. All participants were non-obese, non-smoking adults aged 19–50 years	PRAL_R_: −39⋅0 ± 29⋅0 in strict vegans and −46⋅5 ± 29⋅6 in moderate vegans NEAP_F_: 16⋅3 ± 6⋅73 in strict vegans and −12⋅6 ± 7⋅46 in moderate vegans NEAP_R_: 2⋅41 ± 29⋅3 in strict vegans and −6⋅19 ± 30⋅01 in moderate vegans	Secondary data analysis from the German Vegan Study Calcium was not included in the algorithm
Knurick *et al.*^(134)^	United States of America	*n* 82 participants, thereof *n* 27 meat eaters, *n* 27 lacto-ovo-vegetarians and *n* 28 vegans. All participants were non-obese, non-smoking adults aged 19–50 years with at least 1 year of dietary adherence	PRAL_R_: −15⋅2 ± 40⋅5 in vegans, −1⋅5 ± 23⋅9 in vegetarians and 19⋅6 ± 24⋅3 in omnivores	NEAP_F_ and NEAP_R_ were not determined as part of the study
Storz *et al.*^(12)^	United States of America	*n* 191 self-perceived lacto-ovo-vegetarians aged 18 years or older	PRAL_R_: −0⋅44 (−12⋅19 to 11⋅01) NEAP_F_: 39⋅60 (31⋅48 to 52⋅07) NEAP_R_: 41⋅30 (28⋅63 to 52⋅49)	Secondary data analysis using data from the National Health and Nutrition Examination Surveys

DAL, dietary acid load; NEAP, net endogenous acid production; PRAL, potential renal acid load.

**Table 5.** Dietary intervention studies investigating DAL scores in plant-based study populations

Study (year)	Location	Participants	Results	Comments
Cosgrove and Johnston^(32)^	United States of America	*n* 23 participants, thereof *n* 7 individuals on a vegan diet for 2 d over 1 week (VEG2), *n* 8 individuals on a vegan diet for 3 d over 1 week (VEG3) and *n* 8 individuals on a vegan diet for 7 consecutive days (VEG7)	PRAL_R_ fell from 18⋅1 ± 10⋅07 to 5⋅3 ± 11⋅4 in the combined VEG2/VEG3 group PRAL_R_ fell from 23⋅7 ± 16⋅7 to −6⋅0 ± 12⋅8 in the VEG7 group	Randomised-controlled trial NEAP_F_ and NEAP_R_ were not determined as part of the study Analysis combined (VEG2 and VEG3)
Müller *et al.*^(132)^	Germany	*n* 45 omnivorous individuals randomly assigned to a vegan diet (*n* 23) or a meat-rich diet (*n* 22) for 4 weeks	PRAL_R_ fell from −5⋅26 ± 4⋅45 to −23⋅57 (23⋅87) in vegans after 3 weeks NEAP_F_ fell from fell from 39⋅11(16⋅45) to 24⋅39 ± 7⋅1 in vegans NEAP_R_ fell from 37⋅45 ± 15⋅73 to 12⋅85 ± 19⋅71 in vegans	*Post-hoc* analysis of a randomised-controlled trial All DAL scores increased significantly on a meat-rich diet
Kahleova *et al.*^(14)^	United States of America	*n* 244 participants were randomly assigned to an intervention (vegan) (*n* 122) or control group (*n* 122) for 16 weeks	PRAL_R_ fell from 3⋅6 (0⋅4–6⋅8) to −20⋅7 (−23⋅3 to −18⋅1) in the vegan group NEAP_F_ fell from 50⋅8 (47⋅1–54⋅5) to 25⋅7 (24⋅0–27⋅4) in the vegan group	Secondary data analysis of a randomised clinical trial The authors investigated the effects of an *ad libitum* low-fat vegan diet restricting total fat intake to 10 % of calories No significant changes in the control group

DAL, dietary acid load; NEAP, net endogenous acid production; PRAL, potential renal acid load.

#### Observational studies

We identified four observational studies investigating DAL scores in plant-based individuals^(12,23,133,134)^. Three studies investigated lacto-ovo-vegetarians^(12,133,134)^ and two studies also investigated vegans^(23,134)^. The study characteristics may be obtained in a chronological order from Table 4. All studies found negative PRAL values in individuals consuming a plant-based diet, indicating alkalising properties. The lowest PRAL_R_-values were found in a study by Ströhle *et al.* investigating DAL scores in German vegans (Table 4)^(23)^. Notably, the authors used a modified PRAL_R_ formula and omitted calcium in their calculations.

A Belgian study by Deriemaeker *et al.* also found negative PRAL_R_ scores in vegetarians (−10⋅9 ± 19⋅7 mEq/d)^(133)^, however, their diets were less alkalising as compared with the vegans in Ströhle *et al.*^(23)^. Storz *et al.* performed a secondary data analysis using data from the National Health and Nutrition Examination Surveys^(12)^. The authors investigated DAL scores in self-identified vegetarians who admitted to occasionally consumed animal products^(135)^. Although median PRAL_R_ scores were much higher than in the aforementioned studies, they were still negative (−0⋅44 (−12⋅19 to 11⋅01) mEq/d), also indicating slight alkalising properties.

Generally speaking, vegan diets were associated with lower DAL scores than lacto-ovo-vegetarian diets in all retrieved studies (Table 4). One conceivable explanation is that lacto-ovo-vegetarian diets, which build around eggs, cheese and other dairy products, are usually richer in phosphorus and preservative phosphate (phosphoric acid, polyphosphates) than vegan diets^(132,136)^. Preservative phosphates are characterised by higher gastrointestinal absorption rates and therefore increase the acid load burden from diet^(137)^. We purport that this is one potential factor why vegan diets contribute lower DAL scores than vegetarian diets. An additional difference between these diets is the amino acid composition from protein sources. Protein sources in vegetarian diets include dairy products and/or eggs, which have a greater abundance of sulphur-containing amino acids compared with plant-based protein.

Several large epidemiological investigations suggested that total protein intake is lower in vegan diets as compared with lacto-ovo-vegetarian diets^(138)^. Vegan diets are not deficient in protein but contain significantly higher amounts of plant-based protein^(139)^. One example is the French NutriNet-Santé Study, where vegans consumed on average 12⋅7 g more plant protein per day than vegetarians (46⋅5 g/d *v*. 33⋅8 g/d)^(139)^. This translates into a substantially higher intake of vegetables, fruits and legumes, which generally have alkalising effects^(132)^. The higher the fruits and vegetable intake, the higher the supply of organic anions^(1)^ and thus the higher the alkalising effect of the diet. A reservation must be made, that the protein intake difference between vegans and vegetarians reported in other studies^(140)^ was not as pronounced, possibly due to geographical and socioeconomic factors known to influence nutrition.

#### Clinical intervention studies

We also identified several clinical intervention studies that investigated the effects of various PBDs on DAL management^(14,32,132)^ (Table 5). However, in light of the low number of studies in this field, and with regard to the high heterogeneity in diet composition and study designs, we refrained from performing a meta-analysis.

Cosgrove and Johnston examined the impact of adherence to a vegan diet on acid–base balance in health adults^(32)^. In a randomised-controlled trial, they compared three different diets: a vegan diet for 2 d over 1 week (VEG2), a vegan diet for 3 d over 1 week (VEG3), and a vegan diet for 7 consecutive days (VEG7). With regard to the PRAL-lowering effect, the VEG7 diet performed best. After seven consecutive days on a strict vegan diet, mean PRAL values fell substantially from 23⋅7 ± 17⋅7 to −6⋅0 ± 12⋅8 mEq/d. Again, a strict vegan diet yielded alkalising effects. The effect of the other two dietary interventions (VEG2 and VEG3) was less pronounced.

Our group performed a secondary data analysis of a randomised-controlled trial where 45 omnivorous individuals were randomly assigned to either a vegan diet (*n* 23) or a meat-rich diet (*n* 22) for 4 weeks^(132)^. After 3 weeks, PRAL_R_ scores fell from −5⋅26 ± 4⋅45 to −23⋅57 (23⋅87) mEq/d in vegans. Comparable values were observed in week 4. Notably, the control group comprised individuals on a meat-rich diet, which demonstrated a significant increase in their DAL scores. PRAL_R_ scores rose from 3⋅26 ± 17⋅91 to 18⋅78 (21⋅04) mEq/d in individuals on a meat-rich diet. The isocaloric nature of the vegan diet (participants were instructed to avoid weight loss due to a decreased energy intake) deserves special consideration in this context and might have led to underestimations of the PRAL-lowering effect of vegan diets.

Another important study in the field has been conducted by Kahleova *et al.* in 2021^(14)^. The authors performed a *post-hoc* analysis of a low-fat vegan dietary intervention that restricted processed foods and reduced fat intake to approximately 10 % of total energy. This diet included grains, legumes, vegetables and fruits and was characterised by a targeted macronutrient distribution of approximately 75 % of energy from carbohydrates, 15 % protein and 10 % fat. After 4 months, median PRAL_R_ scores and NEAP_F_ scores dropped significantly in the vegan group (−24⋅3 (−28 to −20⋅5) mEq/d and −25⋅1 (−29⋅1 to −21⋅1) mEq/d, respectively), whereas both scores remained almost identical in the control group (Table 4).

A vegan diet significantly reduced DAL scores in all three studies, however, results from these studies also suggest that dietary adherence is a crucial factor. The simple implementation of one or two ‘vegan days’ per week may be insufficient to achieve an alkalising diet.

## Discussion

There is mounting evidence that PBDs (vegetarian or vegan) may be an effective means to reduce DAL. Observational and clinical studies suggest that both can have alkalising effects, although a vegan diet seems most effective. One limitation is that the total amount of studies in this particular field is still limited and that a direct large-scale randomised-controlled study comparing both diets head-to-head is not yet available. Additional research is thus necessary to identify and quantify the factors that appear to make the vegan diet more favourable towards DAL reduction.

The heterogeneity in studies (and dietary interventions) did not allow us to perform a meta-analysis. Although it is desirable to quantify the PRAL-lowering effects of PBDs, our findings strongly suggest that a vegan diet is associated with an alkaline dietary character, whereas vegetarian diets have rather neutral total PRAL values.

Another point of concern is the lack of a defined reference range for PRAL values and the fact that studies comprised heterogeneous study populations across the world. Depending on sex, age and total energy intake, different reference values may be outlined. Although most studies found lower PRAL values in older adults (potentially due to their lower protein and total energy intake^(12)^, this is not univocally the case)^(141)^. We purport that age is an underestimated factor and suggest that future studies should carefully adjust for that. This might be of particular importance with regard to a potentially progressive loss of bicarbonate in older age^(142)^.

Since neither PRAL nor NEAP scores consider protein origin *per sé* (e.g. animal *v*. plant-based protein, and the corresponding bioavailability of cations and anions contributing to DAL), it would be interesting to examine whether this factor should be incorporated to delineate diet-specific reference ranges of PRAL and NEAP scores to assess and compare DAL more accurately among individuals adhering to different dietary patterns.

In addition to that, future studies should also investigate whether there are potential adverse effects of an overly alkalising diet. According to Xu *et al.*^(143)^, excess diet alkalinity and acidity both showed weak associations with higher mortality in Swedish adults. Comparable findings have been reported in an Iranian study by Hejazi *et al.*^(144)^. Although alkaline diets have been associated with numerous health benefits, we believe that more research is warranted in this area. Quantifying nutrient intake in alkaline diets in comparison with established dietary guidelines would be desirable. A quantification of the effect of colon-produced organic anions and their weighted contribution to DAL would also open a new area of research that has received insufficient attention in the past.

Finally, it is noteworthy that with the ongoing international promotion of plant-based nutrition and the strong growth of food manufacturing of plant-based products, there is a greater consumption of (non-dairy) plant-based cheese alternatives and meat substitutes is also increasing^(145)^. Numerous plant-based cheese alternatives based on nuts, oils, grains, soy and other plant products have been developed – yet their effect on DAL is basically unexplored. The traditional PRAL tables usually date back to over to decades^(20)^, and do not index these new products. A first attempt in this context has been made by Deriemaeker *et al.* who quantified the PRAL values of typical products consumed by vegetarians (in mEq/100 g)^(133)^ (Table 6). Additional research in this area is warranted to better understand the impact of those ‘relatively new’ foods on DAL.

**Table 6.** Selected PRAL values of typical products consumed by vegetarians *v*. non-vegetarians (in mEq/100 g): an overview

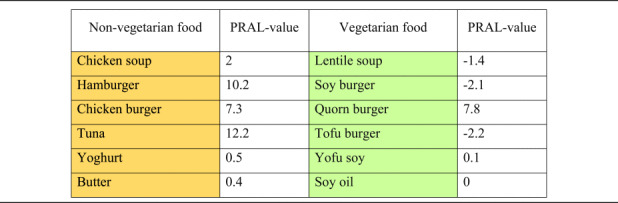

PRAL, potential renal acid load.

Orange colouring: animal-based foods, green colouring: plant-based foods.

PRAL values modified from^(133)^.

## Conclusion

Multiple observational and clinical studies suggest that vegetarian and vegan diets are an effective means to reduce DAL. The vegan diet in particular appears to have alkalising effects and might be more effective than a vegetarian diet to lower PRAL-scores. The lower content of phosphorus, total protein and sulphur-containing amino acids and the abundance of potassium salts from organic anions makes this dietary pattern particularly effective. Additional trials are warranted to understand the impact of the various plant-based dietary patterns on DAL. In this context, it is also of paramount importance to better understand the impact of plant-based cheese and meat alternatives, which are based on nuts, oils, grains, soy and other plant products.
